# Validation of using gene expression in mononuclear cells as a marker for hepatic cholesterol metabolism

**DOI:** 10.1186/1476-511X-5-22

**Published:** 2006-08-15

**Authors:** Dimple Aggarwal, Hedley C Freake, Ghada A Soliman, Amrita Dutta, Maria-Luz Fernandez

**Affiliations:** 1 Department of Nutritional Sciences, University of Connecticut, Storrs, CT 06269, USA; 2 Division of Metabolism, Endocrinology and Diabetes, Department of Internal Medicine, University of Michigan Medical School Ann Arbor, Michigan, 48109, USA

## Abstract

HMG-CoA reductase and the LDL receptor are ubiquitously expressed in major tissues. Since the liver plays a major role in regulating circulating LDL, it is usually of interest to measure the effects of drug or dietary interventions on these proteins in liver. In humans, peripheral blood mononuclear cells have been used as a surrogate for liver to assess regulation of these genes, although there is concern regarding the validity of this approach. The purpose of this study was to evaluate the relationship between liver and mononuclear cell expression of HMG-CoA reductase and the LDL receptor in guinea pigs, a well established model for human cholesterol and lipoprotein metabolism. We extracted RNA from liver and mononuclear cells of guinea pigs from a previous study where the effects of rapamycin, an immunosuppresant drug used for transplant patients, on lipid metabolism were evaluated. Guinea pigs were assigned to three different diets containing the same amount of fat (15 g/100 g) and cholesterol (0.08 g/100 g) for a period of 3 weeks. The only difference among diets was the concentration of rapamycin: 0, 0.0028 or 0.028 g/100 g. There were no differences in plasma LDL cholesterol (LDL-C) among groups. Values were 78.4 ± 14.3, 65.8 ± 17.2 and 68.4 ± 45.4 mg/dL (P > 0.05) for guinea pigs treated with 0, low or high doses of rapamycin, respectively. The mRNA abundance for the LDL receptor and HMG-CoA reductase was measured both in liver (n = 30) and mononuclear cells (n = 22) using reverse transcriptase PCR. In agreement with the finding of no changes in plasma LDL-C, there were also no differences for the expression of HMG-CoA reductase or the LDL receptor among groups. However, a positive correlation was found between liver and mononuclear cells for both HMG-CoA reductase (r = 0.613, P < 0.01) and the LDL receptor (r = 0.622, P < 0.01). These correlations suggest that monocytes can be used in humans as an index for liver to assess diet and drug effects on the expression of HMG-CoA reductase and the LDL receptor.

## Findings

Although cholesterol is an extremely important biological molecule with a major role in membrane structure as well as a precursor for the synthesis of steroid hormones and bile acids, excessive cholesterol, is involved in atherosclerotic lesions. Therefore, a balance must be maintained between cholesterol absorption, excretion and endogenous cholesterol synthesis. In this regard, the liver plays an important role in controlling the amount and composition of circulating LDL cholesterol (LDL-C) levels [1]. For example, under increased dietary cholesterol challenge, the LDL receptor, responsible for the uptake of LDL-C is downregulated [1]. Under similar conditions the rate limiting enzyme of cholesterol synthesis, 3-hydroxy-3methyl glutaryl Coenzyme A (HMG-CoA) reductase is also downregulated [2] and at the same time cholesterol 7α-hydroxylase (CYP7), responsible for catabolism of cholesterol as bile, is upregulated [3] as part of a compensatory mechanism in the liver. Experiments aimed towards the evaluation of drug or dietary interventions conducted in animals rely highly on the liver to evaluate major mechanisms responsible for alterations of plasma lipids. Unfortunately, the liver is not readily accessible during clinical trials. In various human studies, mononuclear cells have been reported to be used as surrogates for liver to estimate hepatic expression of various genes involved in cholesterol metabolism; however, the extent to which mononuclear cells reflect hepatic expression can be questioned [4,5]. Major proteins involved in cholesterol metabolism present in liver are also ubiquitously expressed in other tissues. HMG-CoA reductase and the LDL receptor are clear examples of such proteins. The main purpose of this study was the validation of gene expression in mononuclear cells as marker of hepatic cholesterol metabolism. Guinea pigs were used as the animal model because of their well documented similarities to humans in terms of cholesterol and lipoprotein metabolism [6]. In addition, previous studies performed in our laboratory have reported that guinea pigs serve as a good model for evaluating diet and drugs affecting lipid metabolism [6,7]. We used liver and mononuclear cells that were available from guinea pigs treated with rapamycin where no effects were seen on plasma LDL cholesterol. Thus we were not expecting an effect on LDL receptor or HMG-CoA reductase expression due to treatment [8].

## Methods

### Diets and animals

Diets were designed to meet the nutritional requirements of guinea pigs. All diets contained the same amount of fat, 15 g/100 g and of dietary cholesterol, 0.08 g/100 g and they only varied in the amount of rapamycin (0, 0.0028 or 0.028 g/100 g). Thirty male guinea pigs (Sprague Dawley, Elm Hills Lab) (n = 10 per group) were used for this experiment [8]. Guinea pigs consumed the diets for 3 weeks and diets were weighed daily to determine the amount of food consumed. Guinea pigs were deprived of food overnight and sacrificed by heart puncture after isoflurane anesthesia. Blood was obtained via heart puncture. After euthanasia, the liver was removed, wrapped in labeled, precooled aluminum foil, and then snap frozen in liquid nitrogen and stored immediately at -80°C until the day of RNA extraction. Experimental protocols were approved by the University of Connecticut Institutional Animal Care and Use Committee.

### Mononuclear cell isolation

Mononuclear cells were isolated from whole blood by centrifugation on a Ficoll gradient by the method of Boyum [9]. Briefly, 20 ml blood was diluted with 10 ml HBSS without Ca^2+ ^and Mg^2+^, layered over 10 ml Histopaque^® ^1077 and centrifuged at 500 × g for 30 minutes. The mononuclear cell interface was removed, and washed with HBBS and centrifuged at 600 × g for 10 minutes twice. The cell pellet was resuspended in 200 μl Tris buffer (10 mM Tris, 150 mM NaCl, 1 mM CaCl_2_, pH 7.4), and kept at -80°C until RNA was extracted.

### RNA extraction

Total RNA was extracted from the liver and mononuclear cells by the method of Chomczynski et al. [10]. To check the integrity of the extracted RNA, samples were electrophoresed through a 1% agarose gel at 125 V for 45 minutes. Two clear sharp bands represented 28 S and 18 S ribosomal fraction of RNA (data not shown).

#### RNA quantification

HMG-CoA reductase and LDL receptor mRNA abundance were determined from liver and mononuclear cell extracts using a semi-quantitative RT-PCR method adapted from that of Powell and Kroon [11]. The RT-PCR reaction was carried out using a Qiagen One Step RTPCR kit in a Gene Amp^® ^PCR system 9700 (Applied Biosystems, CA) thermal cycler. Oligonucleotide primers used for amplification were those used for HMG-CoA reductase [12] and the LDL receptor in humans [13]. The primers used for β-actin, a house keeping gene, were taken from guinea pig sequences and have been previously reported [14]. The approximate size of each reaction product is as follows: HMG-CoA reductase : 255 bp, LDL receptor : 240, and β-actin: 220 bp. β-Actin was used as a control in all reactions. The reaction mixture contained 1.0 μg of total RNA. Amplification was carried out at an annealing temperature of 56° for 35 cycles for HMG-CoA reductase, 56° for LDL receptor for 32 cycles, 56° for β-actin for 27 cycles. Ten μL of each reaction mixture was size fractionated by electrophoresis in a 1% agarose gel, in 1% Tris-borate/EDTA buffer. Bands were visualized by staining with ethidium bromide. The amplified RT-PCR products were electrophoresed through a 1% agarose gel at 125 V for 45 minutes. Band sizes were identified using a DNA molecular marker.

Products were then quantified by measuring the relative band intensity using The Image J program (NIH). Band intensities were corrected based on the β-actin signal.

## Results

**Figure 1:**
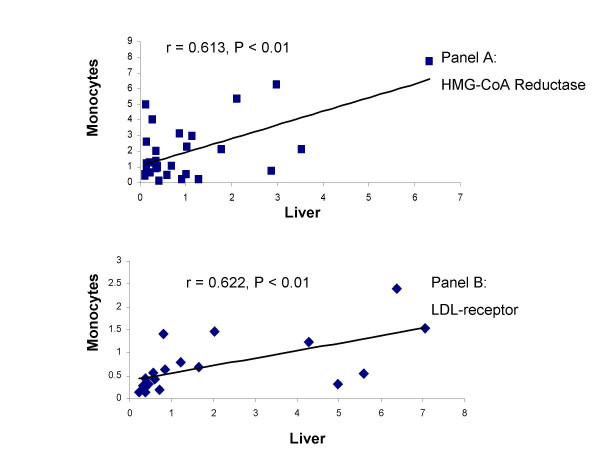
(Panel A) Correlation between liver and mononuclear cells for the expression of HMG-CoA reductase (n = 30). (Panel B) Correlation between liver and mononuclear cells for the expression of the LDL receptor (n = 22).

There were no differences in plasma cholesterol among groups although there were individual differences in guinea pigs ranging from 34 to 173 mg/dL. Mean values were 78.4 ± 14.3, 65.8 ± 17.2 and 68.4 ± 45.4 mg/dL for the 0, low and high rapamycin, respectively. In agreement with these data, there were no significant differences in the expression of the LDL receptor or HMG-CoA reductase among groups. The relative values for mononuclear cell HMG-CoA reductase expression were 2.61 ± 2.33, 1.95 ± 1.85 and 1.39 ± 1.52 for control, low and high rapamacyn, respectively while the corresponding values for the liver were 1.54 ± 1.90, 0.81 ± 0.91 and 0.77 ± 1.04. Similarly, there was no treatment effect for the LDL receptor. The relative values for 0, low and high rapamycin in the liver were 1.13 ± 1.24, 4.6 ± 4.96 and 2.09 ± 1.91 and the corresponding values in the mononuclear cells were 1.21 ± 0.75, 1.64 ± 2.24, and 1.62 ± 3.33. A strong positive correlation was found between the expression of HMG-CoA reductase in the liver and in the mononuclear cells (r = 0.613, P < 0.01) (Figure 1, panel A). The expression of LDL receptor in liver and mononuclear cells were also positively correlated (r = 0.623, P < 0.01) (Figure 1, Panel B).

## Discussion

The current study provided an opportunity to study gene expression in both liver and mononuclear cells to validate mononuclear cells as surrogates of hepatic expression of HMG-CoA reductase and the LDL receptor.

The liver being a biologically active organ is often harvested to study the activity and expression of various proteins affected by dietary interventions or drug treatment [2,8]. Analysis of hepatic enzymes or receptors is feasible in animal studies but it becomes a limiting factor in human studies thus the use of mononuclear cells becomes a desirable option. However, there has always been a concern whether the generated data truly represent hepatic activity. However, there is one study conducted in humans which demonstrated that the mechanisms which regulate mRNA levels in liver and mononuclear cells are similar and suggested that mononuclear cells can be used to predict HMG-CoA reductase and LDL receptor mRNA levels in liver [15]. The functionality of the LDL receptor has been shown in mononuclear cells in which the concentration of both protein and mRNA were evaluated in subjects who had been treated with plant stanol [16]. Another study reported an increased expression of HMG-CoA reductase for human mononuclear cells in subjects consuming 10 g/d of psyllium for one month [4], results which are in agreement with hepatic HMG-CoA reductase activity being up-regulated in guinea pigs after psyllium consumption [17]. In addition, previous studies in our laboratory have shown that the decreases in LDL-C observed following a weight reduction program were correlated with increased expression of the LDL receptor in human mononuclear cells [5].

The positive correlation found between the expression of the LDL receptor and HMG-CoA reductase in liver and mononuclear cells in the current study supports the measurement of the expression of these two ubiquitously distributed proteins in human mononuclear cells as a surrogate for hepatic cholesterol metabolism.

## Abbreviations

HMG-CoA: 3-hydroxy-3methyl glutaryl Coenzyme A; LDL-C: LDL cholesterol; RCT: reverse cholesterol transport

## Competing interests

The author(s) declare that they have no competing interests.

## Authors' contributions

DA did the assays, wrote the manuscript and participated in the interpretation of data; GS: assisted in data interpretation and critical evaluation of manuscript; AD: assisted in the molecular techniques; HF: developed the molecular techniques and assisted in data interpretation and MLF designed the experiment, evaluated the results, interpreted the data and participated in manuscript preparation.
